# ﻿Five new species and one new record of the genus *Phaenocarpa* Foerster (Hymenoptera, Braconidae, Alysiinae) from South Korea

**DOI:** 10.3897/zookeys.1217.129916

**Published:** 2024-11-01

**Authors:** Ju-Hyeong Sohn, Cornelis van Achterberg, Sangjin Kim, Hyojoong Kim

**Affiliations:** 1 Animal Systematics Lab., Department of Biological Science, Kunsan National University, Gunsan, 54150, Republic of Korea Kunsan National University Gunsan Republic of Korea; 2 State Key Laboratory of Rice Biology and Ministry of Agriculture, Key Lab of Agricultural Entomology, Institute of Insect Science, Zhejiang University, Hangzhou, 310058, China Zhejiang University Hangzhou China

**Keywords:** Alysiini, Hymenoptera, new species, parasitoid wasp, phylogeny, taxonomy

## Abstract

Five new species of the genus *Phaenocarpa* Foester, 1863 (Braconidae: Alysiinae) are described and illustrated: *P.acutidentata* Sohn & van Achterberg, **sp. nov.**, *P.tacitoides* Sohn & van Achterberg, **sp. nov.**, *P.setosa* Sohn & van Achterberg, **sp. nov.**, *P.tanycauda* Sohn & van Achterberg, **sp. nov.**, and *P.angusticeps* Sohn & van Achterberg, **sp. nov.** Additionally, *P.tacita* Stelfox, 1941 is recorded for the first time from South Korea. The barcode region of mitochondrial cytochrome c oxidase I (COI) was also analyzed for the seven congeneric species including one from GenBank. In addition, an identification key for the *Phaenocarpa* species recorded in Korea is provided.

## ﻿Introduction

The subfamily Alysiinae is a relatively large taxon in the family Braconidae, consisting of more than 2,440 valid species worldwide (Yu et al. 2016). Alysiinae is subdivided into two tribes, Alysiini and Dacnusini, with 76 and 31 genera, respectively. In South Korea, 270 species in 21 genera have been recorded, 132 within Alysiini and 138 within Dacnusini (NIBR 2022). These two tribes can be distinguished from each other by the presence (Alysiini) or the absence (Dacnusini) of a vein r-m on the fore wing (Shaw and Huddleston 1991). Alysiinae forms part of the Cyclostome clade and comprises exclusively koinobiont endoparasitoids of dipterous larvae (Yu et al. 2016). They use the outwardly curved or straight teeth (with 2–7 teeth) of the exodont mandible to emerge from the host puparium (Docavo et al. 2002). Several species have been utilized for biological control in *Liriomyzatrifolii* (Diptera, Agromyzidae) and *Drosophilasuzukii* (Diptera, Drosophilidae)(Ozawa et al. 2001; Chabert et al. 2012).

The genus *Phaenocarpa* Foerster, 1863 is a large and worldwide genus of Alysiinae, which includes 231 species in nine subgenera (Yu et al. 2016; Zhu et al. 2017). *Phaenocarpa* species are known as koinobiont endoparasitoids, mainly parasitizing larvae of diverse dipterous families, such as Anthomyiidae, Chloropidae, Clusiidae, Drosophilidae, Muscidae, Scathophagidae, Sciomyzidae, Syrphidae, and Muscidae (Wharton 1984; van Achterberg 1998, 2009). This genus can be diagnosed from other alysiine genera by the following combination of characters: the first flagellomere is shorter than the second flagellomere (rarely subequal), vein 3–SR of forewing is longer than vein 2–SR, and vein CU1b is longer than 3–CU1.

In Korea, Papp (1968, 1994) has recorded four species: Phaenocarpa (Discphaenocarpa) angustiptera Papp, 1968, P. (Phaenocarpa) eunice (Haliday, 1838), P. (P.) picinervis (Haliday, 1838), and P. (P.) ruficeps (Nees von Esenbeck, 1812). Later, Sohn et al. (2021) recorded five additional species: Phaenocarpa (P.) artotemporalis Sohn & van Achterberg, 2021, P. (P.) brachyura Sohn & van Achterberg, 2021, P. (P.) lobata Sohn & van Achterberg, 2021, P. (P.) masha Belokobylskij, 1998, and P. (P.) fidelis Fischer, 1970.

In this study, we used the cytochrome c oxidase I (COI), barcode region of the Korean seven *Phaenocarpa* species to confirm their pairwise genetic distances. The comparative diagnoses provides the diagnosis of each new and unrecorded species, comparing them with species that have similar characters. Descriptions, diagnoses, identification keys, and photographs of the diagnostic characters are also provided.

## ﻿Materials and methods

Samples of the new species were collected using Malaise traps in South Korea at Mt. Odaesan (Gangwon-do), Mt. Kalbong (Gyeonggi-do), and Gwaneumsa Temple (Jeju-do). The sorting and preparation were performed at the Animal Systematics Laboratory (**ASL**), Department of Biological Science, Kunsan National University (**KSNU**).

Wharton et al. (1997) and Zhu et al. (2017) were used for morphological identification of generic and subgeneric levels. Morphological characters were observed with a Leica M205 C stereomicroscope. The Taxapad database (Yu et al. 2016) was used for references up to 2015. The terminology follows Wharton (2002) and van Achterberg (1993). Holotypes of the new species have been deposited in the **NIBR** (National Institute of Biological Resources, Incheon) collection. A Leica DMC2900 digital camera and a Leica M205 C stereo microscope (Leica Geosystems AG, Mannheim, Germany) were used for photography; all pictures were taken for each final photo using multi-focusing technology. LAS V4.11 (Leica Geosystems AG, St. Gallen, Switzerland) and Helicon Focus 7 (Helicon Soft, Kharkiv, Ukraine) software were used to stack the photographs. Final illustrations were created using Adobe Photoshop CS6.

For DNA analyses, whole genomic DNA was extracted from the specimens using a Labopass Tissue kit (Cosmo Genetech, Daejeon, Korea) following the manufacturer’s protocol. In order to conserve morphologically complete voucher specimens, the ‘non-destructive method’ by Favret (2005) and the ‘freezing method’ by Yaakop et al. (2009) were used, with slight modification to avoid the first crushing of the sample. In the original protocol, the sample was crushed or damaged and then soaked in 180 μL of buffer ATL + 20 μL of proteinase and incubated at 55 °C for 3 h. In the slightly modified DNA extraction methods, samples were incubated in 180 μL of buffer ATL + 20 μL of proteinase K without first crushing the sample, followed by a 10-min incubation at 55 °C and then kept in a freezer at −22 °C overnight. Subsequently, a general protocol was followed for the remaining steps. The primer sets of *LCO-1490* (5’-GGTCAACAAATCATAAAGATATTGG-3’) and HCO-2198 (5’-TAAACTTCAGGGTGACCAAAAAATCA-3’) was used to amplify approximately 658 bp as the partial front region of the COI. The polymerase chain reaction (PCR) products were amplified by using AccuPowerH PCR PreMix (BIONEER, Corp., Daejeon) in 20 μl reaction mixtures containing 0.4 μM of each primer, 20 μM of the dNTPs, 20 μM of the MgCl_2_, and 0.05 μg of the genomic DNA template. PCR amplification was performed using a GS1 thermo-cycler (Gene Technologies, Ltd., U.K) according to the following procedure: initial denaturation at 95 °C for 5 min, followed by 34 cycles at 94 °C for 35 sec; an annealing temperature of 48 °C for 25 sec; an extension at 72 °C for 45 sec, and a final extension at 72 °C for 5 min. PCR products were visualized using electrophoresis and a 1.5% agarose gel. A single band was observed and sequenced using an automated sequencer (ABI Prism 3730 XL DNA Analyzer, California, USA) at Macrogen Inc. (Seoul, South Korea).

## ﻿Results

### ﻿COI analysis

A total of 586 bp of the COI fragments were sequenced from *P.tacita* Stelfox, *P.acutidentata* sp. nov., *P.tacitoides* sp. nov., *P.setosa* sp. nov., *P.tanycauda* sp. nov., and *P.angusticeps* sp. nov. that were deposited in GenBank (accession numbers PP587250–PP587256) (Table 1). Pairwise distances were estimated by using the *P*-distance model with the option for pairwise deletion. Interspecific distance ranged from 0.053 to 0.268 (average 0.131) (Table 2).

**Table 1.** Species list for COI analysis for the present study.

No	Species	NCBI accession number	Reference
1	*P.acutidentata* Sohn & van Achterberg, sp. nov.	PP587250	this study
2	*P.angusticeps* Sohn & van Achterberg, sp. nov.	PP587255	this study
3	*P.artotemporalis* Sohn & van Achterberg, 2021	MZ318086	Sohn et al. 2021
4	*P.brachyura* Sohn & van Achterberg, 2021	MZ318087	Sohn et al. 2021
5	*P.fidelis* Fischer, 1970	MZ318083	Sohn et al. 2021
6	*P.lobata* Sohn & van Achterberg, 2021	MZ318085	Sohn et al. 2021
7	*P.masha* Sohn & van Achterberg, 2021	MW376066	Sohn et al. 2021
8	*P.ruficeps* (Nees, 1812)	MZ318084	Sohn et al. 2021
9	*P.setosa* Sohn & van Achterberg, sp. nov.	PP587252	this study
10	*P.tacita* Stelfox, 1941	PP587256	this study
11	*P.tacitoides* Sohn & van Achterberg, sp. nov.	PP587251	this study
12	*P.tanycauda* Sohn & van Achterberg, sp. nov.	PP587254	this study

**Table 2.** Calculated genetics distance, based on COI sequences between *Phaenocarpa* species used in the analysis.

	*P.acutidentata*	*P.angusticeps*	*P.artotemporalis*	*P.brachyura*	*P.fidelis*	*P.lobata*	*P.masha*	*P.ruficeps*	*P.setosa*	*P.tacita*	*P.tacitoides*	*P.tanycauda*
*P.acutidentata*												
*P.angusticeps*	0.111											
*P.artotemporalis*	0.162	0.140										
*P.brachyura*	0.106	0.108	0.123									
*P.fidelis*	0.104	0.108	0.125	0.072								
*P.lobata*	0.097	0.104	0.128	0.072	0.053							
*P.masha*	0.135	0.135	0.152	0.137	0.123	0.126						
*P.ruficeps*	0.114	0.099	0.140	0.108	0.097	0.087	0.121					
*P.setosa*	0.099	0.114	0.147	0.094	0.094	0.094	0.125	0.101				
*P.tacita*	0.119	0.113	0.109	0.118	0.106	0.108	0.121	0.104	0.108			
*P.tacitoides*	0.104	0.104	0.133	0.096	0.096	0.108	0.126	0.097	0.099	0.108		
*P.tanycauda*	0.241	0.249	0.268	0.234	0.237	0.234	0.242	0.247	0.229	0.241	0.251	

### ﻿Taxonomy

#### ﻿ Phaenocarpa

Taxon classificationAnimaliaHymenopteraBraconidae

Foerster, 1863

A10E4CA1-7E70-5CF8-8C6A-F44AE38A7C7F

Phaenocarpa Foerster, 1863: 267. Type species (by original designation): Alysiapicinervis Haliday, 1838.

##### Diagnosis.

Third antennal segment shorter than fourth segment; fore wing vein 2–SR shorter than vein 3–SR, vein CU1b longer than vein 3–CU1; vein 1-M of hind wing comparatively long.

##### Biology.

Koinobiont endoparasitoids of larvae of Dipteran species (Wharton 1984).

##### Distribution.

Cosmopolitan.

### ﻿Key to the Korean *Phaenocarpa* species

**Table:** 

1	Temples distinctly striate ventrally; mesopleuron largely coarsely sculptured; face laterally extensively and finely striate	***P.angustiptera* Papp, 1968**
–	Temples smooth ventrally; mesopleuron largely smooth, except for area of precoxal sulcus; face laterally smooth or nearly so	**2**
2	Vein r-m of fore wing bordered with blackish setae, resulting in one infuscated patch; mesoscutum with metallic sheen	***P.picinervis* (Haliday, 1838)**
–	Vein r-m of fore wing normal, not bordered with blackish setae; mesoscutum without metallic sheen	**3**
3	Mandible slender, ~ 2.3 × longer than wide; vein 2-1A of fore wing of ♂ strongly widened	***P.eunice* (Haliday, 1838)**
–	Mandible robust, at most 1.9 × longer than wide; vein 2-1A of fore wing of ♂ narrow	**4**
4	Scutellar sulcus evenly narrowed medially, 3–5 × wider than its median length; head more or less reddish or yellowish brown	***P.ruficeps* (Nees, 1812)**
–	Scutellar sulcus wide medially, 2–3 × wider than long medially; head dark brown or black	**5**
5	First tooth of mandible gradually connected to second tooth, forming a straight or arcuate connection (Figs 3K, 4K)	**6**
–	First tooth of mandible separated from second tooth by small incision (Figs 1K, 2K, 5K, 6K)	**9**
6	Ovipositor sheath approx. as long as hind tibia; first tergite 1.6–2.0 × longer than its apical width	**7**
–	Ovipositor sheath 1.4 × longer than hind tibia; first tergite at most 1.5 × longer than its apical width	**8**
7	First flagellomere ~ 2 × longer than wide; second flagellomere 1.6 × longer than first flagellomere; notauli not reaching medio-posterior depression; vein 3-SR 1.6 × longer than 2-SR; antenna without whit segments	***P.fidelis* Fischer, 1970**
8	Second flagellomere 2 × longer than first flagellomere (Fig. 5J); mandible 1.8 × longer than wide (Fig. 4K); first metasomal tergite 1.5 × longer than its apical width (Fig. 4H)	***P.tanycauda* Sohn & van Achterberg, sp. nov.**
–	Second flagellomere 1.4 × longer than first flagellomere (Fig. 3J); madible 1.4 × longer than wide (Fig. 3K); first metasomal tergite as long as wide apically (Fig. 3H)	***P.setosa* Sohn & van Achterberg, sp. nov.**
9	First flagellomere 2.8–3.6 × longer than wide	**10**
–	First flagellomere 4.2–5.1 × longer than wide (Figs 1J, 2J, 5J, 6J)	**13**
10	Eye in dorsal view 4.0–4.5 × as long as temple; propleuron reddish brown; notauli reduced posteriorly	***P.artotemporalis* Sohn & van Achterberg, 2021**
–	Eye in dorsal view 2.0–3.0 × as long as temple; propleuron black or orange-brown; notauli usually complete (up to medio-posterior depression or nearly so)	**11**
11	Mandible subparallel-sided; setose part of ovipositor sheath ~ 0.7 × as long as hind tibia; first metasomal tergite ~ 1.4 × longer than its apical width	***P.brachyura* Sohn & van Achterberg, 2021**
–	Mandible distinctly widened dorsally; setose part of ovipositor sheath 1.2–1.3 × as long as hind tibia; first tergite 1.1–1.2 × longer than its apical width	**12**
12	Tarsal claws slender; third and fourth antennal segments dark brown and slender; metanotum more or less tooth-shaped protruding dorsally in lateral view; pterostigma ~ 4 × longer than wide; middle tooth of mandible not widened dorsally	***P.masha* Belokobylskij, 1998**
–	Tarsal claws robust; third and fourth antennal segments yellow and robust; metanotum obtuse dorsally in lateral view; pterostigma ~ 5.5 × longer than wide; middle tooth of mandible widened dorsally	***P.lobata* Sohn & van Achterberg, 2021**
13	Precoxal sulcus distinct and complete, reaching anterior and posterior edge of mesopleuron, (Fig. 1G); ovipositor sheath 1.9 × longer than hind tibia; second flagellomere 1.5 × longer than first flagellomere; propodeum entirely rugose	***P.acutidentata* Sohn & van Achterberg, sp. nov.**
–	Precoxal sulcus only medially impressed, not reaching anterior and posterior edge of mesopleuron (Fig. 2J, 5J, 6J); ovipositor sheath 0.8–1.0 × as long as hind tibia; second flagellomere 1.1–1.2 × longer than first flagellomere; propodeum entirely smooth except for medio-longitudinal carina	**14**
14	Mandible 1.2 × longer than its maximum width (Fig. 5K); minimum width of face (Fig. 5E) 0.9 × its height (measured from ventral rim of antennal sockets to upper margin of clypeus); first flagellomere 4.3 × longer than wide (Fig. 5J)	***P.angusticeps* Sohn & van Achterberg, sp. nov.**
–	Mandible 1.6 × longer than its maximum width (Fig. 2K, 6K); minimum width of face (Fig. 2E, 6E) 1.4 × its height (measured from ventral rim of antennal sockets to upper margin of clypeus); first flagellomere 4.9–5.1 × longer than wide	**15**
15	Vein r of fore wing short, ~ 1.4 × longer than wide, vein SR1 of fore wing straight (Fig. 2C); hind femur more slender, 5.6 × longer than width; first tooth of mandible lobe-shaped, widened dorsally, 1.7 × as long as third; [apical antennal segments paler than subbasal segments]	***P.tacitoides* Sohn & van Achterberg, sp. nov.**
–	Vein r of for wing ~ 3.0 × longer than wide, vein SR1 of fore wing slightly curved (Fig. 6C); hind femur 4.6 × longer than width. first tooth of mandible acute and as long as third tooth	***P.tacita* Stelfox, 1941**

#### ﻿ Phaenocarpa acutidentata

Taxon classificationAnimaliaHymenopteraBraconidae

Sohn & van Achterberg sp. nov.

1CEC2932-1225-51BF-AAE3-A13BA22B3A2A

https://zoobank.org/7F346053-E46C-4CA6-A821-3DA7A288AE39

Fig. 1

##### Type material.

***Holotype*** • ♀ (NIBR), **South Korea**, Mt. Odae, Jinbu-myeon, Pyeongchang-gun, Gangwon-do, 37°45'54.7"N, 128°34'13.8"E, 15.IX.2020, Ju-Hyeong Sohn leg. GenBank accession no. PP587250.

##### Comparative diagnosis.

The new species is similar with *P.telengai* Belokobylskij, 1998, but recognizable the third tooth distinctly and acutely protruding as the first one (only second tooth narrow and acute in *P.telengai*), first flagellomere 4.2 × longer than wide (2.8–3.0 × in *P.telengai*) and hind femur 4.2 × longer than wide (4.7–5.0 × in *P.telengai*).

##### Description.

Holotype, ♀, body 2.8 mm in lateral view, fore wing 2.6 mm, ovipositor sheath 1.4 mm in lateral view, antenna 3.4 mm (apical part of antennae missing).

**Figure 1.**
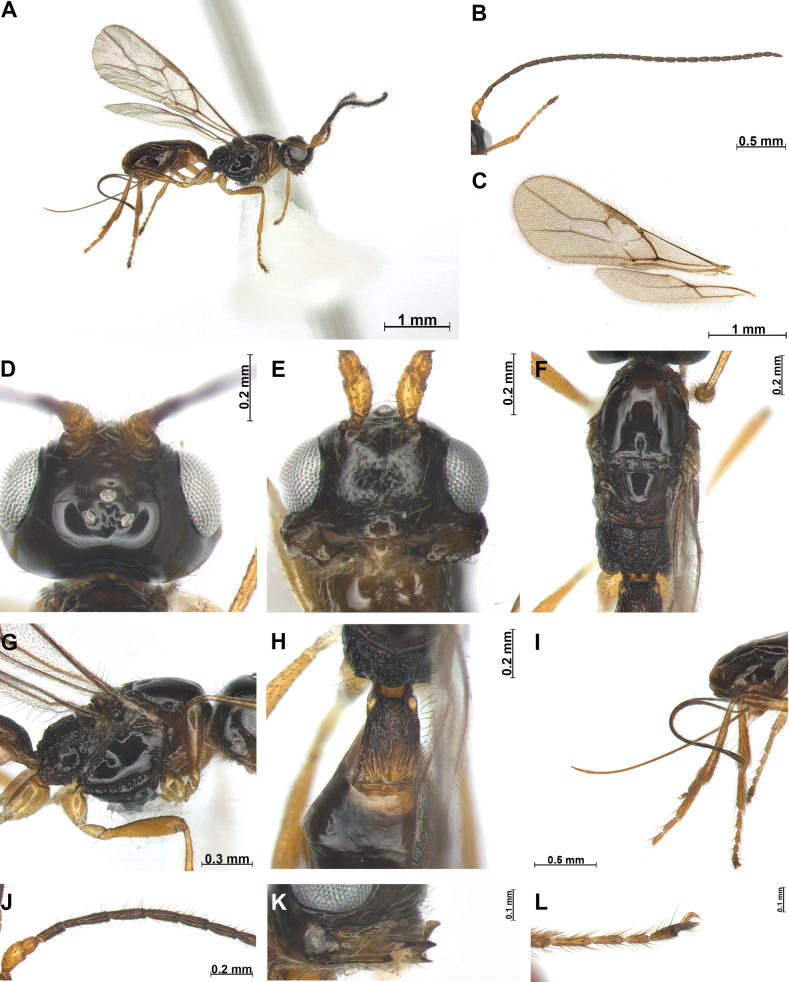
*Phaenocarpaacutidentata* sp. nov., ♀ **A** habitus, lateral view **B** antennae **C** wings **D** head, dorsal view **E** head, frontal view **F** mesosoma, dorsal view **G** mesosoma, lateral view **H** first metasomal tergite, dorsal view **I** ovipositor sheath, lateral view **J** proximal part of antenna **K** mandible, lateral view **L** claw, lateral view.

***Head***: Width of head 1.5 × its median length in dorsal view (Fig. 1D). Antenna with 25 antennomeres (terminal antennomere missing), first flagellomere 4.2 × longer than wide (Fig. 1K), second flagellomere 1.5 × longer than first antennomere and 5.2 × longer than wide. Medial antennal segments (18^th^ segment) 2.3 × longer than wide. Eye slightly oval, in lateral view 1.2 × as long as wide. Minimum width of face (Fig. 1E) 1.2 × its height; face rugose with setose. Eye in dorsal view 1.8 × as long as temple. Ocello-ocular line (OOL) 4.8 × longer than diameter of anterior ocellus; OOL: antero-posterior ocellar line (AOL): postero-ocellar line (POL) = 30: 7: 10. Vertex smooth and glabrous. Width of clypeus 2.3 × its maximum length. Mandible (Fig. 1L) 1.8 × longer than wide, wide with first tooth curved; second tooth narrow and long, 1.2 × longer than first tooth, tip of second tooth reddish brown; third tooth (as first) distinctly acutely protruding; carina on third tooth distinct.

***Mesosoma***: In dorsal view mesosoma 2.2 × longer than wide 1.6 × longer than high in lateral view. Mesoscutum (Fig. 1F) with small and round medio-posterior depression and glabrous; notauli impressed anteriorly, not reaching to medio-posterior depression; scutellar sulcus with one carina; apical part of mesopleuron and metapleuron without setae, posterior mesopleural groove sculpted. Precoxal sulcus distinct, reaching anterior and posterior edge of mesopleuron. Maximum length of propodeum (Fig. 1F) 0.5 × as long as its width, largely sculpted; posterior part of propodeum with small areola, not reaching middle of propodeum. Metanotum not protruding medio-dorsally in lateral view (Fig. 1G). Fore wing (Fig. 1C) 2.6 × as long as wide; pterostigma widened medially 5.1 × longer than wide; vein r of fore wing 4.0 × longer than wide; vein SR1 1.8 × longer than vein 3-SR; vein 3-SR 1.5 × longer than 2-SR; second submarginal cell long and narrow, 2.7 × longer than its medium length; 3-SR: r: SR1 = 12: 3: 22; first discal cell of fore wing 0.8 × longer than wide in median length; first discal cell of fore wing 1.1 × as longer medially than wide. Hind wing: vein M+CU+1-M: vein 1r-m = 4: 1.

***Leg***: Hind femur 4.2 × longer than wide and 0.7 × as long as hind tibia; hind tibia 11 × longer than wide; hind tibia as long as hind tarsus.

***Metasoma***: First tergite (Fig. 1H) sparsely rugose, 1.5 × longer than its apical width. Setose part of ovipositor sheath (Fig. 1I) 1.4 × longer than mesosoma and 1.9 × longer than hind tibia.

***Color***: Body (Fig. 1A) dark brown, antenna dark brown, but anterior yellowish brown; metasoma dark brown; legs yellowish brown; first tergite brown.

**Male.** Unknown.

##### Biology.

Unknown.

##### Host.

Unknown.

##### Distribution.

South Korea.

##### Etymology.

The specific name *acutidentata* is an adjective, referring to ‘sharp teeth’ in Latin.

#### ﻿ Phaenocarpa tacitoides

Taxon classificationAnimaliaHymenopteraBraconidae

Sohn & van Achterberg sp. nov.

DB47EB49-49F3-59EB-91BE-58B7F6C7D14F

https://zoobank.org/AF3679A0-77B0-44DC-B3A1-1C4D994019C6

Fig. 2

##### Type material.

***Holotype*** • ♀ (NIBR), **South Korea**, Mt. Kalbong, Gyeongban-ri, Gapyeong-eup, Gapyeong-gun, Gyeonggi-do, 37°51'10.9"N, 127°26'27.4"E, 11.V.2020, Sohn. GenBank accession no. PP587251.

##### Comparative diagnosis.

Differs from the other *Phaenocarpa* species by vein r of fore wing being much more distal (1.4 × longer than wide). This species similar with *P.tacita* Stelfox, 1941, but easily distinguish by apical antennal segments paler than subbasal segments, and first tooth of mandible lobe-shaped, widened dorsally, 1.7 × as long as third (first tooth of mandible acute and as long as third tooth in *P.tacita*).

##### Description.

Holotype, ♀, body 1.7 mm in lateral view, fore wing 2.0 mm, ovipositor sheath 0.5 mm, antenna 1.5 mm.

**Figure 2.**
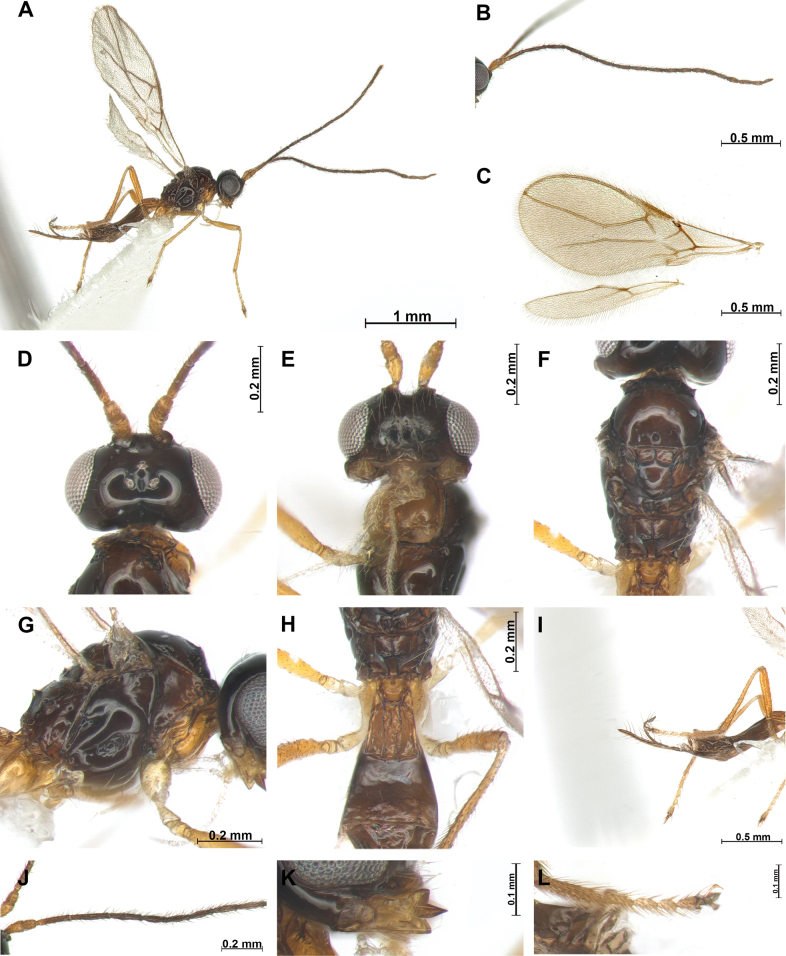
*Phaenocarpatacitoides* sp. nov., ♀ **A** habitus, lateral view **B** antennae **C** wings **D** head, dorsal view **E** head, frontal view **F** mesosoma, dorsal view **G** mesosoma, lateral view **H** first metasomal tergite, dorsal view **I** ovipositor sheath, lateral view **J** proximal part of antenna **K** mandible, lateral view **L** claw, lateral view.

***Head***: Width of head 1.7 × its median length in dorsal view (Fig. 2D). Antenna with 18 antennomeres, first flagellomere slender, 4.9 × longer than wide (Fig. 2K), second flagellomere 1.2 × longer than first and 6.1 × longer than wide. Medial antennal segments 4.1 × longer than wide. Apical antennal segments paler than subbasal segments. Eye slightly oval, in lateral view 1.1 × as long as wide. Minimum width of face (Fig. 2E) 1.4 × its height; face rugose with setose. Eye in dorsal view 2.5 × as long as temple. Ocello-ocular line (OOL) 5.3 × longer than diameter of anterior ocellus; OOL: antero-posterior ocellar line (AOL): postero-ocellar line (POL) = 21: 5: 6. Vertex smooth and glabrous. Width of clypeus 2.2 × its maximum length. Mandible (Fig. 2L) 1.6 × longer than wide, wide with first tooth slightly curved upward; second tooth narrow and long, 1.2 × longer than first tooth, tip of second tooth reddish brown; third tooth as long as first tooth, carina on third tooth distinct.

***Mesosoma***: In dorsal view mesosoma 1.8 × longer than wide and 1.4 × longer than high in lateral view. Mesoscutum (Fig. 2F) with small and round medio-posterior depression and glabrous; notauli impressed anteriorly, not reaching medio-posterior depression; mesoscutum without setae; scutellar sulcus with one carina; apical part of mesopleuron and metapleuron without setae, posterior mesopleuron groove smooth. Precoxal sulcus distinct, but not reaching anterior and posterior edge of mesopleuron. Maximum length of propodeum (Fig. 2F) 0.6 × its width; medio–longitudinal carina present on half of propodeum, posterior part of propodeum with areola, reaching middle of propodeum. In lateral view metanotum with acute protuberance medio-dorsally (Fig. 2G). Fore wing (Fig. 2C) 2.3 × as long as wide; pterostigma widened medially 6.2 × longer than wide; vein r of fore wing 1.4 × longer than wide; vein SR1 2.1 × longer than vein 3-SR; vein 3-SR 1.8 × longer than 2-SR; second submarginal cell long and narrow, 2.8 × longer than its medium length; 3-SR: r: SR1 = 15: 2: 33; first discal cell of fore wing ~ 1.1 × as long medially as wide. Hind wing: vein M+CU+1-M: vein 1r-m = 5: 1.

***Leg***: Hind femur slender, 5.6 × longer than wide and 0.6 × as long as hind tibia; hind tibia 19 × longer than wide; hind tibia as long as hind tarsus.

***Metasoma***: First tergite (Fig. 2H) medially rugose and 1.5 × longer than its apical width. Setose part of ovipositor sheath (Fig. 2I) as long as than mesosoma and as long as hind tibia.

***Color***: Body (Fig. 2A) brown; head dark brown; first tergite yellowish brown, metasoma brown; antenna dark brown; legs yellowish brown.

**Male.** Unknown.

##### Biology.

Unknown.

##### Host.

Unknown.

##### Distribution.

South Korea.

##### Etymology.

The specific name *tacitoides* is an adjective, named after *P.tacita* and -*oides* added a suffix because of its similarity to this species (“oides” is Latin for “resembling”).

#### ﻿ Phaenocarpa setosa

Taxon classificationAnimaliaHymenopteraBraconidae

Sohn & van Achterberg sp. nov.

49338940-19C3-5C45-B7F0-73948337AA31

https://zoobank.org/7CB6ABB9-FEF2-4EEB-A9F1-A5980EE615C1

Fig. 3

##### Type material.

***Holotype*** • ♀ (NIBR), **South Korea**, Mt. Kalbong, Gyeongban-ri, Gapyeong-eup, Gapyeong-gun, Gyeonggi-do, 37°51'10.9"N, 127°26'27.4"E, 05.VI.2020, Sohn. GenBank accession no. PP587252.

##### Comparative diagnosis.

**Figure 3.**
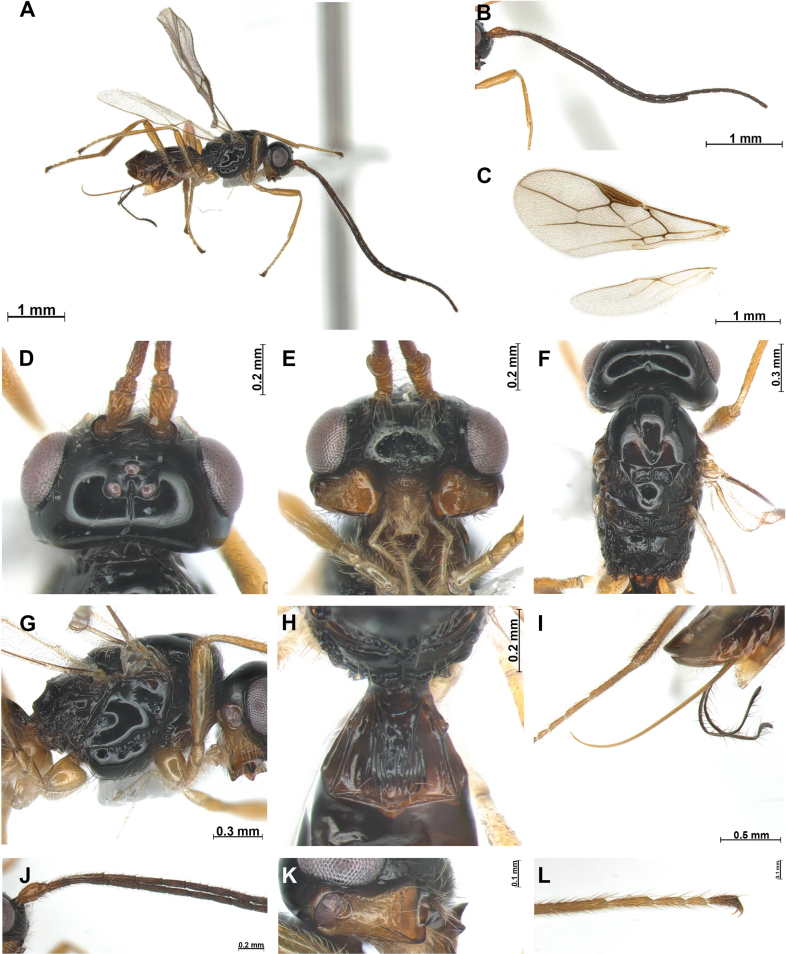
*Phaenocarpasetosa* sp. nov., ♀ **A** habitus, lateral view **B** antennae **C** wings **D** head, dorsal view **E** head, frontal view **F** mesosoma, dorsal view **G** mesosoma, lateral view **H** first metasomal tergite, dorsal view **I** ovipositor sheath, lateral view **J** proximal part of antenna **K** mandible, lateral view **L** claw, lateral view.

This new species is close to *P.micula* Belokobylskij, 1998, because of sharing width of the first flagellomere (3.0–3.5 × in *P.micula*), deep and smooth notauli and width of the first tergite. However, the new species has the metanotum not protruding (tooth-like protruding in *P.micula*) (Fig. 3G), upper tooth of mandible separated from middle tooth (not separated in *P.micula*), lower tooth of mandible angulate (rounded in *P.micula*), hind tibia partly erect setose (Fig. 3A), width of head 1.7 × greater than its median length (2.0–2.2 × in *P.micula*), second flagellomere 2.0 × longer than first flagellomere (1.5–1.6 × in *P.micula*).

##### Description.

Holotype, ♀, body 2.8 mm in lateral view, fore wing 2.7 mm, ovipositor sheath 1.3 mm, antenna 3.8 mm (but apical parts missing).

***Head***: Width of head 1.7 × its median length in dorsal view (Fig. 3D). Antenna with 23 antennomeres (but apical parts missing), first flagellomere 2.9 × longer than wide (Fig. 3K), second flagellomere 2.0 × longer than first and 7.3 × longer than wide. Medial antennal segments 2.5 × longer than wide. Eye slightly oval, in lateral view 1.2 × as long as wide. Minimum width of face (Fig. 3E) 1.6 × its height; face setose, upper part of clypeus rugose. Eye in dorsal view 1.6 × as long as temple. Ocello-ocular line (OOL) 4.8 × longer than diameter of anterior ocellus; OOL: antero-posterior ocellar line (AOL): postero-ocellar line (POL) = 14: 3: 4. Vertex smooth and glabrous, with distinct longitudinal groove. Width of clypeus 2.6 × its maximum length. Mandible (Fig. 3L) 1.4 × longer than wide, wide with first tooth curved upward and separated from second tooth, second tooth robust, 1.1 × longer than first tooth, tip of second tooth dark brown; third tooth as long as first tooth, not protruding and angulate apically, carina on third tooth distinct.

***Mesosoma***: In dorsal view mesosoma 2.0 × longer than wide and 1.4 × longer than high in lateral view. Mesoscutum (Fig. 3F) with small and round medio-posterior depression and glabrous; notauli distinctly impressed, almost reaching medio-posterior depression; mesoscutum without setae; scutellar sulcus with one carina; apical part of metapleuron with setae, posterior mesopleuron groove sculptured. Precoxal sulcus distinct, reaching at anterior and posterior edge of mesopleuron. Maximum length of propodeum (Fig. 3F) 0.8 × its width longitudinal carina present on half of propodeum, posterior part of propodeum with areola, reaching up to half of propodeum. In lateral view metanotum obtuse curved medio-dorsally (Fig. 3G). Fore wing (Fig. 3C) 2.3 × as long as wide; pterostigma widened medially 4.5 × longer than wide; vein r of fore wing 1.3 × longer than wide; vein SR1 2.1 × longer than vein 3-SR; vein 3-SR 1.2 × longer than 2-SR; second submarginal 2.1 × longer than its medium length; 3-SR: r: SR1 = 11: 1: 25; first discal cell of fore wing ~ 1.3 × longer medially than wide. Hind wing: vein M+CU+1-M: vein 1r-m = 3: 1.

***Leg***: Hind femur 5.7 × longer than wide and 0.8 × as long as hind tibia; hind tibia 10 × longer than wide and setae erect except basally (Fig. 3A); hind tibia as long as hind tarsus.

***Metasoma***: First tergite (Fig. 3H) medially rugose and as long as its apical width. Setose part of ovipositor sheath 1.2 × longer than mesosoma (Fig. 3I) and 1.4 × longer than hind tibia.

***Color***: Body (Fig. 3A) black; metasoma and antenna dark brown; legs reddish brown.

**Male.** Unknown.

##### Biology.

Unknown.

##### Host.

Unknown.

##### Distribution.

South Korea.

##### Etymology.

The specific name *setos*” is an adjective, named after the erect setae of the hind tibia.

#### ﻿ Phaenocarpa tanycauda

Taxon classificationAnimaliaHymenopteraBraconidae

Sohn & van Achterberg sp. nov.

1865E0A2-0308-5CCD-8456-F359EE96DBFB

https://zoobank.org/80C76B8C-AB95-48F0-A03C-5928187AA520

Fig. 4

##### Type material.

***Holotype*** • ♀ (NIBR), **South Korea**, Mt. Kalbong, Gyeongban-ri, Gapyeong-eup, Gapyeong-gun, Gyeonggi-do, 37°51'10.9"N, 127°26'27.4"E, 05.VI.2020, Sohn. GenBank accession no. PP587254.

##### Comparative diagnosis.

This new species is close to *P.chasanica* Belokobylskij, 1998 because of sharing second flagellomere 1.4–1.5 × longer than first flagellomere (same length in *P.chasanica*). Width of head 1.8 × its median length in dorsal view (1.8–2.0 × in *P.chasanica*). In mandible, first and second tooth not separated distinctly. Hind femur 5.0–5.5 × as long as wide. The new species differs from *P.chasanica* by head and mesosoma black (head and mesosoma yellowish brown in *P.chasanica*) and thick and short tarsal claws (tarsal claws thin and relatively long in *P.chasanica*). The new species can be recognised by its comparatively long ovipositor sheath (1.2 × longer than mesosoma and 1.4 × longer than hind tibia) and rugose median part of face rugose.

##### Description.

Holotype, ♀, body 2.5 mm in lateral view, fore wing 2.5 mm, ovipositor sheath 1.0 mm, antenna 3.9 mm.

**Figure 4.**
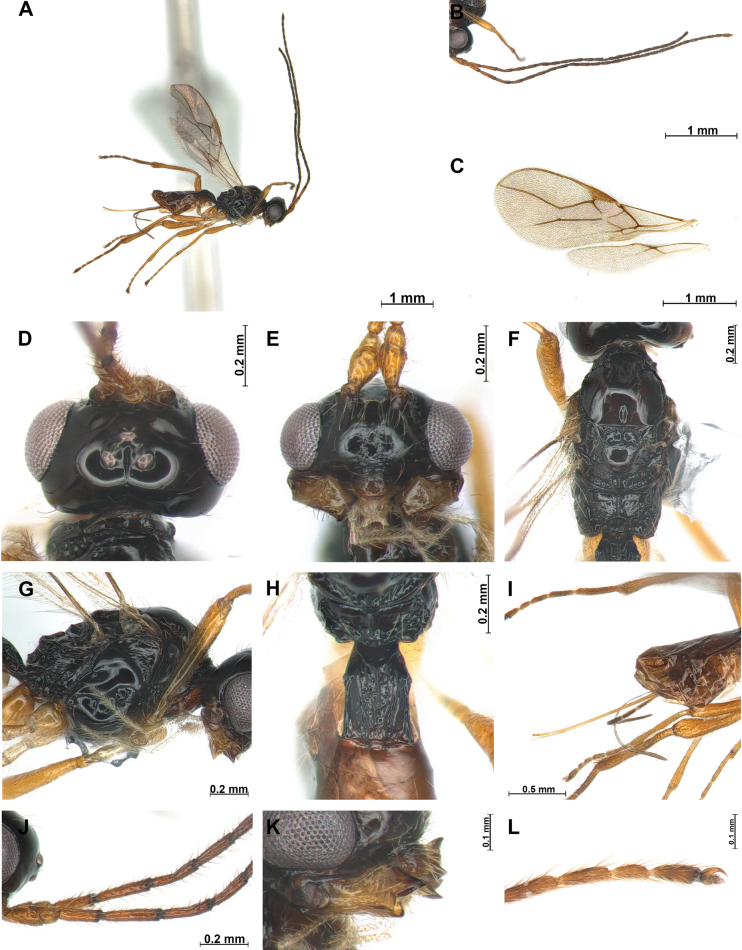
*Phaenocarpatanycauda* sp. nov., ♀ **A** habitus, lateral view **B** antennae **C** wings **D** head, dorsal view **E** head, frontal view **F** mesosoma, dorsal view **G** mesosoma, lateral view **H** first metasomal tergite, dorsal view **I** ovipositor sheath, lateral view **J** proximal part of antenna **K** mandible, lateral view **L** claw, lateral view.

***Head***: Width of head 1.8 × its median length in dorsal view (Fig. 4D). Antenna with 26 antennomeres and 1.6 × as long as fore wing or body. First flagellomere 3.2 × longer than wide (Fig. 4K). Second flagellomere 1.4 × longer than first and 4.8 × longer than wide. Middle of antenna segment 3.1 × longer than width. Eye slightly oval, in lateral view 1.1 × as long as wide. Minimum width of face (Fig. 4E) 1.3 × its height; face with setose, median part of face rugose. Eye in dorsal view 2.6 × as long as temple. Ocello-ocular line (OOL) 4.2 × longer than diameter of anterior ocellus; OOL: antero-posterior ocellar line (AOL): postero-ocellar line (POL) = 27: 8: 9. Vertex smooth and glabrous, short longitudinal groove present POL. Width of clypeus 2.6 × its maximum length. Mandible (Fig. 4L) 1.8 × longer than wide, wide with first tooth curved upward and broad; second tooth long and broad, 1.3 × longer than first tooth, tip of second tooth reddish brown; first and second tooth not separate distinctly; third tooth as long as first tooth.

***Mesosoma***: In dorsal view mesosoma 1.9 × longer than wide and 1.4 × longer than high in lateral view. Mesoscutum (Fig. 4F) with small and round medio-posterior depression and glabrous; notauli distinct anteriorly to half of mesoscutum, not reaching to medio-posterior depression; mesoscutum few setae along notauli; scutellar sulcus with one strong carina; apical part of mesopleuron and metapleuron with few setae, mesopleural groove sculptured. Precoxal sulcus distinct, reaching anterior and posterior edges of mesopleuron. Maximum length of propodeum (Fig. 4F) 0.5 × its width; longitudinal carina present on propodeum, posterior part of propodeum with small areola, not reaching to half of propodeum. In lateral view, metanotum not protruding medio-dorsally (Fig. 4G). Fore wing (Fig. 4C) 2.4 × as long as wide; pterostigma broad, 4.3 × longer than wide; vein r of fore wing 1.7 × longer than wide; vein SR1 2.1 × longer than vein 3-SR; vein 3-SR 1.5 × longer than 2-SR; second submarginal cell 2.6 × longer than its medium length; 3-SR: r: SR1 = 13: 1: 27 first discal cell of fore wing as long as wide medially. Hind wing: vein M+CU+1-M: vein 1r-m = 4: 1.

***Leg***: Hind femur 5.4 × as long as wide and 0.7 × as long as hind tibia; hind tibia 8.8 × longer than wide; hind tibia 0.8 × as long as hind tarsus.

***Metasoma***: First tergite (Fig. 4H) rugose 1.4 × longer than its apical width. Setose part of ovipositor sheath (Fig. 4I) 1.2 × longer than mesosoma, 1.4 × longer than hind tibia and 0.4 × as long as fore wing.

***Color***: Body (Fig. 4A) black; metasoma (except first tergite) and antenna dark brown but basal part of antenna and legs yellowish brown.

**Male.** Unknown.

##### Biology.

Unknown.

##### Host.

Unknown.

##### Distribution.

South Korea.

##### Etymology.

The specific name *tanycauda* is an adjective, named after the long ovipositor sheath; *tanyo* is Greek for stretched out, *cauda* is Latin for tail.

#### ﻿ Phaenocarpa angusticeps

Taxon classificationAnimaliaHymenopteraBraconidae

Sohn & van Achterberg sp. nov.

411C83D8-74C7-5693-834C-F02298A6944E

https://zoobank.org/998AAEBA-48AD-4866-BE3C-B0AFFF07A756

Fig. 5

##### Type material.

***Holotype*** • ♀ (NIBR), **South Korea**, Gwaneumsa, Sanrokbuk-ro, Jeju-si, Jeju-do, 33°25'43.9"N, 126°33'24.8"E, 06.VII.2020, Sohn. GenBank accession no. PP587255.

##### Comparative diagnosis.

Differs from all the species of *Phaenocarpa* by having narrow face, 0.9 × from ventral rim of antennal sockets to upper margin of clypeus (1.2–1.6 × in other species). Second flagellomere 1.1 × longer than first flagellomere (1.2–2.0 × in other species).

##### Description.

Holotype, ♀, body 2.4 mm in lateral view, fore wing 2.7 mm, ovipositor sheath 0.8 mm, antenna 3.5 mm.

**Figure 5.**
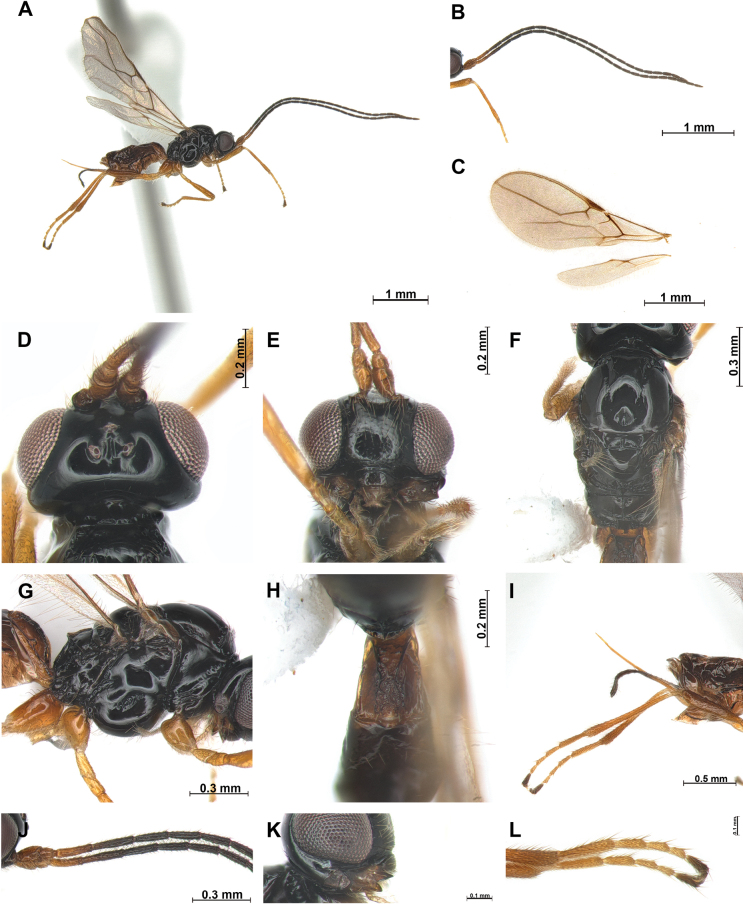
*Phaenocarpaangusticeps* sp. nov., ♀ **A** habitus, lateral view **B** antennae **C** wings **D** head, dorsal view **E** head, frontal view **F** mesosoma, dorsal view **G** mesosoma, lateral view **H** first metasomal tergite, dorsal view **I** ovipositor sheath, lateral view **J** proximal part of antenna **K** mandible, lateral view **L** claw, lateral view.

***Head***: Width of head 1.8 × its median length in dorsal view (Fig. 5D). Antenna with 23 antennomeres. First flagellomere 4.3 × longer than wide (Fig. 5K), second flagellomere 1.1–1.2 × longer than first and 5.3 × longer than wide. Middle of antenna segment 3.4 × longer than wide. Eye slightly oval, in lateral view 1.2 × as long as wide. Minimum width of face (Fig. 5E) 0.9 × its height; face smooth with setose. Eye in dorsal view 2.4 × as long as temple. Ocello-ocular line (OOL) 4.9 × longer than diameter of anterior ocellus; OOL: antero-posterior ocellar line (AOL): postero-ocellar line (POL) = 28: 7: 9. Vertex smooth and glabrous, longitudinal groove present POL. Width of clypeus 2.6 × its maximum length. Mandible (Fig. 5L) 1.2 × longer than wide, wide with first tooth curved upward and broad; second tooth narrow and long, 1.2 × longer than first tooth, tip of second tooth dark brown; first and second tooth distinctly separate; third tooth as long as first tooth.

***Mesosoma***: In dorsal view mesosoma 2.0 × longer than wide and 1.4 × longer than high in lateral view. Mesoscutum (Fig. 5F) with small and round medio-posterior depression and glabrous; notauli distinct anteriorly to half of mesoscutum, not reaching to medio-posterior depression; anterior part of mesoscutum with few setae; scutellar sulcus with one carina; apical part of mesopleuron and metapleuron with few setae, mesopleuron groove sculptured in lateral view. Precoxal sulcus distinct, not reaching anterior and posterior edge of mesopleuron. Maximum length of propodeum (Fig. 5F) 0.5 × its width; longitudinal carina present on propodeum, posterior part of propodeum with small areola, not reaching up to half of propodeum, anterior part of propodeum smooth. In lateral view, metanotum curved medio-dorsally (Fig. 5G). Fore wing (Fig. 5C) 2.3 × as long as wide; pterostigma widened medially and nearly as wide as vein 1-R1; vein r of fore wing 3.8 × longer than wide; vein SR1 3.0 × longer than vein 3-SR; vein 3-SR 1.8 × longer than 2-SR; second submarginal cell 2.4 × longer than its medium length; 3-SR: r: SR1 = 5: 1: 15; first discal cell of fore wing as long as wide medially. Hind wing: vein M+CU+1-M: vein 1r-m = 4: 1.

***Leg***: Hind femur 6.0 × as long as wide and 0.7 × as long as hind tibia; hind tibia 15 × as long as wide; hind tibia 1.1 × longer than hind tarsus.

***Metasoma***: First tergite (Fig. 5H) rugose medially, 1.4 × longer than its apical width. Setose part of ovipositor sheath (Fig. 5I) 0.9 × as long as mesosoma, 0.8 × as long as hind tibia.

***Color***: Body (Fig. 5A) black; head black; first tergite reddish brown, metasoma reddish brown; antenna dark brown, anterior parts yellowish brown, apical parts brown; legs yellowish brown.

**Male.** Unknown.

##### Biology.

Unknown.

##### Host.

Unknown.

##### Distribution.

South Korea.

##### Etymology.

The specific name *angusticeps* is an adjective, *angustus* is Latin for narrow, *ceps* is Latin for head.

#### ﻿ Phaenocarpa tacita

Taxon classificationAnimaliaHymenopteraBraconidae

Stelfox, 1941

36BF99F7-E840-51B4-A995-FCE19566535F

Fig. 6

##### Type material.

***Holotype*** • (NIBR), **South Korea**, Mt. Kalbongsan, Gyeongban-ri, Gapyeong-eup, Gapyeong-gun, Gyeonggi-do, 37°51'10.9"N, 127°26'27.4"E, 05.VI.2020, Sohn. GenBank accession no. PP587256.

##### Comparative diagnosis.

**Figure 6.**
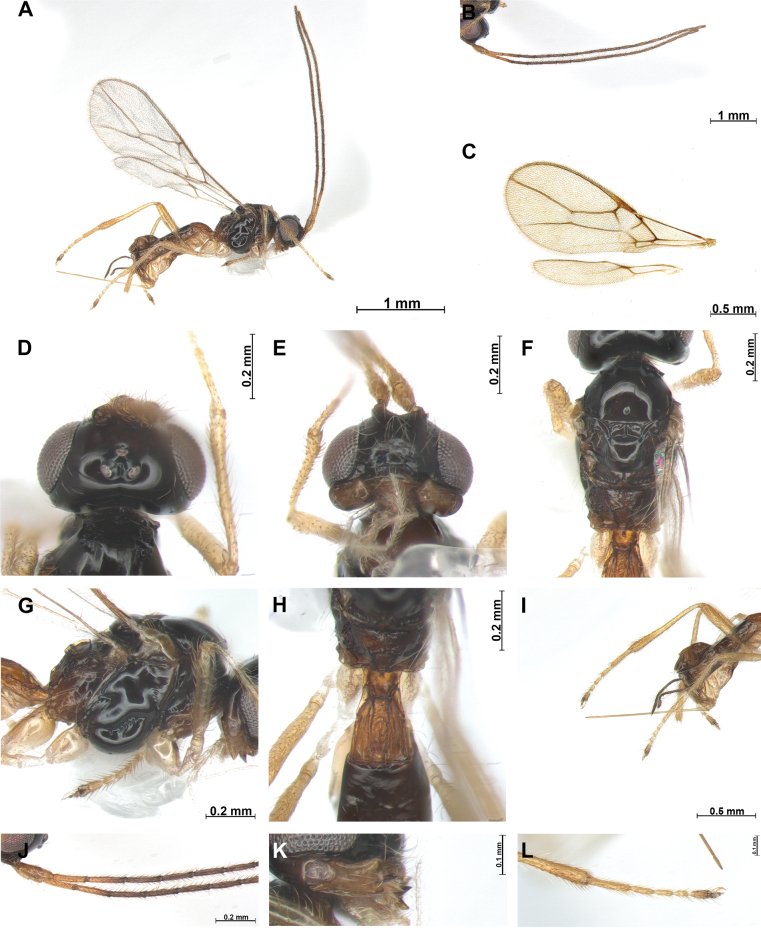
*Phaenocarpatacita* Stelfox, 1941 ♀ **A** habitus, lateral view **B** antennae **C** wings **D** head, dorsal view **E** head, frontal view **F** mesosoma, dorsal view **G** mesosoma, lateral view **H** first metasomal tergite, dorsal view **I** ovipositor sheath, lateral view **J** proximal part of antenna **K** mandible, lateral view **L** claw, lateral view.

According to the East Palaearctic key of Belokobylskij (1998), mandible (Fig. 6K) expanded towards the apical. Propodeum smooth, longitudinal carina present half of the propodeum. First metasomal tergite (Fig. 6H) 1.5 × longer than its apical width (1.4–1.6 × in Belokobylskij 1998). The Korean species ovipositor sheath 0.9 × as long as hind tibia (1.1–1.2 × in Belokobylskij 1998), but according to Belokobylskij (1998), it rarely equals to hind tibia.

##### Description.

♀. Body 1.9 mm in lateral view, fore wing 2.1 mm, ovipositor sheath 0.9 mm, antenna 2.6 mm (apical parts missing).

***Head***: Width of head 1.8 × its median length in dorsal view (Fig. 6D). Antenna with 17 antennomeres (apical parts missing), first flagellomere 5.1 × longer than wide (Fig. 6K), second flagellomere 1.2 × longer than first and 6.1 × longer than wide. Medial antennal segments 4.6 × longer than wide. Eye slightly oval, in lateral view 1.1 × as long as wide. Minimum width of face (Fig. 6E) 1.4 × its height; face setose, upper part of clypeus rugose. Eye in dorsal view 2.3 × as long as temple. Ocello-ocular line (OOL) 4.6 × longer than diameter of anterior ocellus; OOL: antero-posterior ocellar line (AOL): postero-ocellar line (POL) = 22: 6: 7. Vertex smooth and glabrous, with distinct longitudinal groove. Width of clypeus 2.7 × its maximum length. Mandible (Fig. 6L) 1.6 × longer than wide, wide with first tooth curved upward; second tooth narrow and long, 1.3 × longer than first tooth, tip of second tooth dark brown; third tooth as long as first tooth.

***Mesosoma***: In dorsal view mesosoma 2.0 × longer than wide and 1.3 × longer than high in lateral view. Mesoscutum (Fig. 6F) with small and round medio-posterior depression and glabrous; notauli impressed anteriorly, not reaching medio-posterior depression; mesoscutum without setae; scutellar sulcus with one carina; apical part of mesopleuron with few setae, posterior mesopleuron groove sculptured. Precoxal sulcus distinct, but not reaching anterior and posterior edge of mesopleuron. Maximum length of propodeum (Fig. 6F) 0.5 × its width; longitudinal carina present half of propodeum, posterior part of propodeum with areola, reach to half of propodeum. In lateral view, anterior part of metanotum curved medio-dorsally (Fig. 6G). Fore wing (Fig. 6C) 2.3 × as long as wide; pterostigma hardly widened medially and nearly as wide as vein 1-R1; vein r of fore wing 3.0 × longer than wide; vein SR1 1.7 × longer than vein 3-SR; vein 3-SR 2.2 × longer than 2-SR; second submarginal cell 3.3 × longer than its medium length; 3-SR: r: SR1 = 8: 1: 14; first discal cell of fore wing as long as wide medially. Hind wing: vein M+CU+1-M: vein 1r-m = 4: 1.

***Leg***: Hind femur 4.6 × longer than wide and 0.7 × as long as hind tibia; hind tibia 15 × longer than wide; hind tibia as long as hind tarsus.

***Metasoma***: First tergite (Fig. 6H) medially rugose 1.5 × longer than its apical width. Setose part of ovipositor sheath (Fig. 6I) 0.9 × as long as mesosoma and 0.9 × as long as hind tibia.

***Color***: Body (Fig. 6A) dark brown; head dark brown; first tergite reddish brown, metasoma brown; antenna brown, apical parts pale brown; legs pale yellowish brown.

**Male.** Unknown.

##### Biology.

Unknown.

##### Host.

Unknown.

##### Distribution.

Austria (Fischer 1970), Czechoslovakia (Lozan 2004), Hungary (Papp 2008), Ireland (Fischer 1970), Netherlands (van Achterberg 1988), Russia (Belokobylskij 1998), United Kingdom (Kloet and Hincks 1945), new to South Korea.

## ﻿Discussion

The genus *Phaenocarpa* in Korea is a large group with 231 species across nine subgenera, but only nine species were recorded in Korea until now. With the addition of five new and one European species, there are now 15 species of *Phaenocarpa* in Korea. The new species and *Phaenocarpaartotemporalis* Sohn & van Achterberg, 2021, *P.brachyura* Sohn & van Achterberg, 2021, and *P.lobata* Sohn & van Achterberg, 2021 are only recorded in Korea. *Phaenocarpamasha* Belokobylskij, 1998, is recorded in Russia and Korea, and *P.fidelis* Fischer, 1970, *P.angustiptera* Papp, 1968, and *P.eunice* (Haliday, 1838), are recorded in the eastern Palearctic region (Belokobylskij 1998; Sohn et al. 2021). *Phaenocarpapicinervis* (Haliday, 1838) is recorded in both the eastern and western Palearctic regions, and *P.ruficeps* (Nees, 1812) is recorded in all regions except the Neotropic, Arctic, and Australasian regions (Yu et al. 2016).

Unfortunately, all the species used in this study are females, and records of males have not yet been confirmed. However, males could be identified later using COI barcoding and identification keys.

## Supplementary Material

XML Treatment for Phaenocarpa

XML Treatment for Phaenocarpa acutidentata

XML Treatment for Phaenocarpa tacitoides

XML Treatment for Phaenocarpa setosa

XML Treatment for Phaenocarpa tanycauda

XML Treatment for Phaenocarpa angusticeps

XML Treatment for Phaenocarpa tacita

## ﻿Additional information

### Author ORCIDs

Ju-Hyeong Sohn https://orcid.org/0000-0003-0976-4114

Cornelis van Achterberg https://orcid.org/0000-0002-6495-4853

Sangjin Kim https://orcid.org/0000-0001-8395-8019

Hyojoong Kim https://orcid.org/0000-0002-1706-2991

### Data availability

All of the data that support the findings of this study are available in the main text.
